# Identification of a novel stomatal opening chemical, PP242, that inhibits early abscisic acid signal transduction in guard cells

**DOI:** 10.1093/pcp/pcaf013

**Published:** 2025-01-30

**Authors:** Airi Oh, Riku Kimura, Shinpei Inoue, Taiyo Sato, Yuki Hayashi, Ayato Sato, Yohei Takahashi, Toshinori Kinoshita

**Affiliations:** Division of Biological Science, Graduate School of Science, Nagoya University, Chikusa, Nagoya 464-8602, Japan; Division of Biological Science, Graduate School of Science, Nagoya University, Chikusa, Nagoya 464-8602, Japan; Division of Biological Science, Graduate School of Science, Nagoya University, Chikusa, Nagoya 464-8602, Japan; Division of Biological Science, Graduate School of Science, Nagoya University, Chikusa, Nagoya 464-8602, Japan; Division of Biological Science, Graduate School of Science, Nagoya University, Chikusa, Nagoya 464-8602, Japan; Institute of Transformative Bio-Molecules (WPI-ITbM), Nagoya University, Chikusa, Nagoya 464-8601, Japan; Division of Biological Science, Graduate School of Science, Nagoya University, Chikusa, Nagoya 464-8602, Japan; Institute of Transformative Bio-Molecules (WPI-ITbM), Nagoya University, Chikusa, Nagoya 464-8601, Japan; Division of Biological Science, Graduate School of Science, Nagoya University, Chikusa, Nagoya 464-8602, Japan; Institute of Transformative Bio-Molecules (WPI-ITbM), Nagoya University, Chikusa, Nagoya 464-8601, Japan

**Keywords:** abscisic acid signaling, chemical biology, PM H^+^-ATPase, protein phosphorylation, Raf-like kinase, stomatal opening

## Abstract

Plants control their stomatal apertures to optimize carbon dioxide uptake and water loss. Stomata open in response to light through the phosphorylation of the penultimate residue, Thr, of plasma membrane (PM) H^+^-ATPase in guard cells. Stomata close in response to drought and the phytohormone abscisic acid (ABA), and ABA suppresses the light-induced activation of PM H^+^-ATPase. However, the signaling pathways that regulate the stomatal aperture remain unclear. Previously, we identified a target of rapamycin (TOR) inhibitor, temsirolimus, to induce stomatal opening through chemical screening. In the present study, we further investigated other TOR inhibitors and identified PP242 as a novel stomatal opening chemical. PP242 induced stomatal opening even in the dark, as well as phosphorylation of the penultimate Thr of PM H^+^-ATPase in guard cells. Interestingly, PP242 completely suppressed ABA-induced stomatal closure, and inhibited ABA-induced activation of SNF1-related protein kinase 2s (SnRK2s), which are essential kinases for ABA signal transduction in guard cells. *In vitro* biochemical analysis revealed that PP242 did not directly inhibit SnRK2 but rather inhibited upstream ABA-signaling components, specifically B3 clade Raf-like kinases. A quadruple mutant of B3 clade Raf-like kinases exhibited an open stoma phenotype that resembled the effect of PP242. However, PP242 still induced stomatal opening in this mutant, suggesting that PP242 also targets other guard cell components. Together, these results reveal that PP242 induces stomatal opening partly by inhibiting steady-state ABA signal transduction.

## Introduction

Stomata are minute pores surrounded by pairs of guard cells found on the epidermis of terrestrial plants. Stomata open in response to light and close in response to drought and dark. Stomatal opening induces carbon dioxide uptake for photosynthesis and transpiration. Therefore, plants adeptly control stomatal aperture in response to environmental change. Blue light-induced stomatal opening has been thoroughly investigated (Shimazaki et al. 2007, Inoue and Kinoshita 2017). The blue light receptor phototropins phot1 and phot2 play important roles in stomatal movement (Kinoshita et al. 2001, Christie 2007). Blue light-irradiated phototropins autophosphorylate and transmit downstream signals via phosphorylation (Inoue et al. 2008). Phototropin signaling activates plasma membrane (PM) H^+^-ATPase in guard cells. H^+^ extrusion from the cytosol to the apoplast by PM H^+^-ATPase causes membrane hyperpolarization. Subsequently, K^+^ is taken up via inwardly rectifying K^+^ channels and increasing osmotic pressure, which ultimately drives water influx to the guard cells and induces stomatal opening (Shimazaki et al. 2007).

*Arabidopsis thaliana* has 11 PM H^+^-ATPases, among which regulate cell growth and ion homeostasis (Li et al. 2022). The penultimate residue, Thr, of PM H^+^-ATPase (pen-Thr) is critical to the regulation of PM H^+^-ATPase activity (Fuglsang et al. 1999, Svennelid et al. 1999, Kinoshita and Shimazaki 2002). The phosphorylation of pen-Thr causes a formation change of the C terminus, an inhibitory domain, which ultimately activates PM H^+^-ATPase via interaction with 14-3-3 protein. In guard cells, phototropin-mediated blue light signaling induces the phosphorylation of pen-Thr and interaction of 14-3-3 to activate PM H^+^-ATPase (Jahn et al. 1997, Kinoshita and Shimazaki 1999). Thr881 phosphorylation in PM H^+^-ATPase was recently shown to be important for light-induced stomatal opening (Fuji et al. 2024, Hayashi et al. 2024). The fungal toxin fusicoccin (FC) also induces pen-Thr phosphorylation, resulting in irreversible stomatal opening even in the dark. FC enhances the strong interaction between 14-3-3 and the C terminus of PM H^+^-ATPase (Kinoshita and Shimazaki 2001). Thus, regulation of pen-Thr phosphorylation levels is essential for tuning stomatal movement. To date, several signal components have been identified to function in blue light signaling between phototropins and PM H^+^-ATPase in guard cells. For example, the kinase BLUE LIGHT SIGNALING 1 (BLUS1) is a direct substrate for phototropins specific to guard cells (Takemiya et al. 2013). Additionally, BLUE LIGHT-DEPENDENT H^+^-ATPASE PHOSPHORYLATION (BHP) and Type 1 protein phosphatase (PP1) have been proposed to function in blue light signaling in guard cells (Takemiya et al. 2006, Hayashi et al. 2017). However, the entire mechanism of blue light signaling, from phototropins to PM H^+^-ATPase, has not been fully elucidated.

Stomatal closure in response to the phytohormone abscisic acid (ABA) has been extensively studied (Munemasa et al. 2015, Hsu et al. 2021a). ABA signaling activates anion channels such as SLOW ANION CHANNEL-ASSOCIATED 1 (SLAC1) and causes membrane depolarization in guard cells (Vahisalu et al. 2008). This phenomenon ultimately induces a decrease in turgor pressure and stomatal closure. SNF1-related protein kinase 2.6 (SnRK2.6; also called SRK2E/ OST1) plays a critical role in ABA signal transduction in guard cells (Mustilli et al. 2002, Yoshida et al. 2002). B2 and B3 clade Raf-like kinases are known to phosphorylate and activate SnRK2s in *A. thaliana* (Katsuta et al. 2020, Takahashi et al. 2020, Lin et al. 2021, Soma et al. 2023). Under normal conditions, OST1 is suppressed by type 2C protein phosphatase (PP2C) (Umezawa et al. 2009). When ABA receptors (PYL/PYR/RCAR) recognize ABA, they interact with PP2C and suppress its activity. As a result, OST1 autophosphorylates and phosphorylates downstream components, resulting in ABA signal transduction (Hsu et al. 2021a). In addition, ABA signaling inhibits stomatal opening signaling. OST1 suppresses KAT1 channel expression via phosphorylation of ABA-RESPONSIVE KINASE SUBSTRATEs (AKSs), which are basic helix–loop–helix-type transcription factors (Takahashi et al. 2013, 2016). ABA signaling also suppresses PM H^+^-ATPase activity (Goh et al. 1996, Hayashi et al. 2011). Conversely, constitutively activated PM H^+^-ATPase prevents ABA-induced stomatal closure (Merlot et al. 2007). In addition to revealing the entire process of stomatal opening signaling, it is necessary to elucidate the functional mechanisms underlying the crosstalk between multiple signaling pathways, including ABA signal transduction.

Our research group has adopted chemical biology approaches to further elucidate the roles of signaling pathways in stomatal movement. Chemical biology reveals phenotypes that may be hidden in single or partial gene mutations (Park et al. 2009, Hayashi et al. 2017). In our previous study, we discovered that temsirolimus, an inhibitor of target of rapamycin (TOR), acted as a novel stomatal opening inducer (Toh et al. 2018). The chemical regulation of stomatal movement has great potential to increase pathogen resistance and carbon dioxide uptake or regulate drought tolerance, as observed with [5-(3,4-dichlor- ophenyl)furan-2-yl]-piperidine-1-ylmethanethione, (–)-catechin gallate, and benzyl isothiocyanate (Kim et al. 2011, Sato et al. 2022, Aihara et al. 2023).

In this study, we investigated stomatal opening-related chemicals to better understand the roles of signal transduction in guard cells. Building on insights obtained in our previous research on temsirolimus, we investigated TOR inhibitors and identified PP242, a novel stomatal opening inducer. PP242 activated PM H^+^-ATPase via its phosphorylation. Further mechanistic investigation revealed that PP242 inhibited ABA signal transduction in guard cells. These findings elucidate the novel function of PP242 in plants and demonstrate that basal levels of ABA inhibit stomatal opening.

## Results

### Identification of a novel stomatal opening chemical, PP242

**Figure 1.**
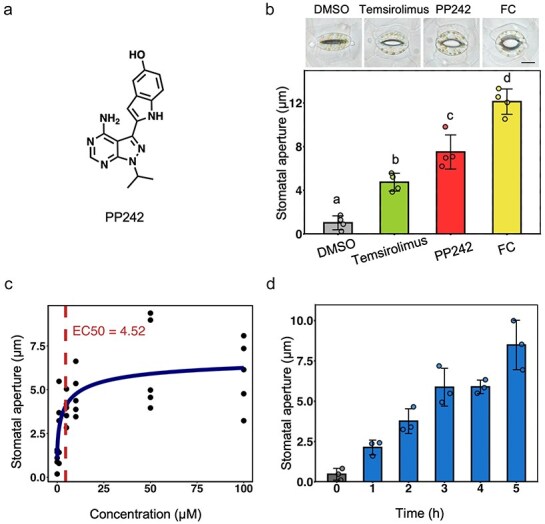
PP242 induces stomatal opening in the epidermis of *Commelina benghalensis*. (a) Structure of PP242. (b) PP242-induced stomatal opening. Images show typical stomatal apertures. Scale bar = 20 µm. Epidermis samples from dark-adapted *C. benghalensis* were treated with 50 µM temsirolimus, 50 µM PP242, or 10 µM fusicoccin (FC) in the dark for 3 h. Data are the means ± standard deviation (SD; *n* = 4; 30 stomata per replicate). Different letters indicate significant differences [*P *< .05; one-way ANOVA followed by Tukey’s honest significant difference (HSD) test]. (c) Dose dependence of stomatal opening in response to PP242. Epidermis samples from dark-adapted *C. benghalensis* were treated for 3 h in the dark. Data are the means of each experiment (*n* = 5; 30 stomata per replicate). (d) Time course of stomatal opening in response to PP242. Epidermis samples from dark-adapted *C. benghalensis* were treated with 50 µM PP242 in the dark. Data are the means ± SD (*n* = 3; 30 stomata per replicate).

Our previous study in *Commelina benghalensis* demonstrated that the TOR inhibitor temsirolimus induces stomatal opening (Toh et al. 2018). Therefore, we investigated whether other TOR inhibitors might also induce stomatal opening, and found that only PP242 and Torin2-induced stomatal opening in *C. benghalensis* in the dark (Fig. 1a and b, Supplementary Fig. S1a and b). PP242-induced stomatal opening was observed in both *A. thaliana* and *Vicia faba* (Supplementary Fig. S2). PP242 has a pyrazolopyrimidine scaffold and an indole (Fig. 1a) (Feldman et al. 2009). Further investigation of the dose dependence and time course of stomatal opening in response to PP242 revealed that the half-maximal effective concentration of PP242-induced stomatal opening was 4.52 µM and that stomatal opening was stable after ∼3 h of treatment in *C. benghalensis* (Fig. 1c and d). These results characterize PP242 as a stomatal opening inducer in plants.

### PP242 activates PM H^+^-ATPase in guard cells

**Figure 2.**
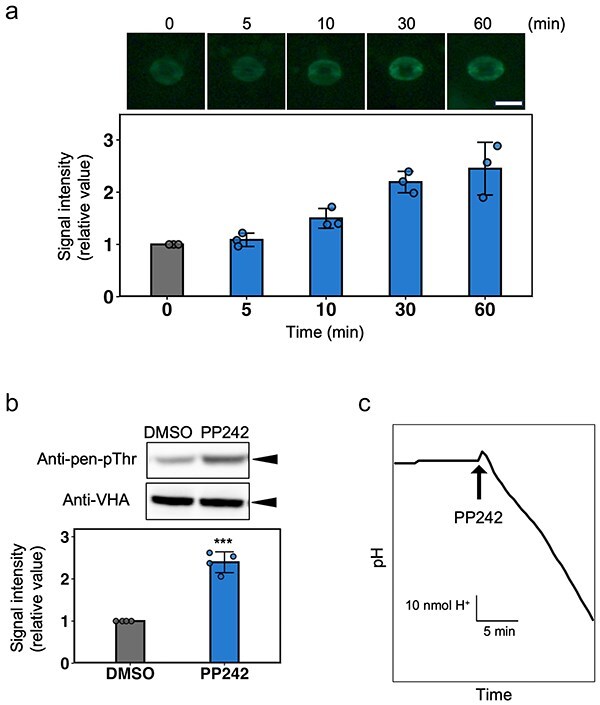
PP242 induces phosphorylation and activation of PM H^+^-ATPase in guard cells. (a) Time course of PP242-induced PM H^+^-ATPase phosphorylation in guard cells. (Upper panel) Typical fluorescent images of stomata using anti-pen-pThr antibody. Epidermis samples from dark-adapted *A. thaliana* wild-type (Col-0; WT) plants were treated with 50 µM PP242 in the dark and incubated for the indicated time. The bar graph shows the quantification of fluorescence intensity. Data are the means ± SD (*n* = 3; fluorescence intensity of 50 stomata per replicate). Scale bar = 20 µm. (b) PP242-induced phosphorylation of PM H^+^-ATPase in guard cell protoplasts (GCPs) of *Vicia faba*. Panels show typical immunoblot images; the bar graph shows band intensity. Dark-adapted GCPs were treated with 50 µM PP242 for 1 h in the dark. Immunoblotting was performed using individual antibodies. Phosphorylation levels of H^+^-ATPase compared to control were determined as band intensity ratios. Data are mean ± SD (*n* = 4). Asterisks indicate significant differences (*P* < .001; Welch’s *t*-test). (c) PP242-dependent H^+^ pumping in GCPs of *V. faba*. H^+^ pumping was determined as the decrease in pH in the medium. GCPs were pre-incubated under red light for 30 min, and then 50 µM PP242 was added to the GCP suspensions. A typical experiment is shown (*n* = 4).

Having identified PP242 as related to stomatal opening, we investigated whether PP242 induces the phosphorylation of pen-Thr of PM H^+^-ATPase, which is indispensable for PM H^+^-ATPase activation (Fuji et al. 2024, Hayashi et al. 2024). Interestingly, PP242 induced pen-Thr phosphorylation in guard cells (Fig. 2a). The phosphorylation level increased after 10 min of treatment, and more than doubled after 30 min (Fig. 2a). PP242 also induced pen-Thr phosphorylation in guard cell protoplasts (GCPs) of *V. faba* (Fig. 2b). Next, we examined the effect of PP242 on PM H^+^-ATPase activity in *V. faba* GCPs. PP242-induced PM H^+^-ATPase-mediated H^+^ extrusion from a GCP suspension (Fig. 2c, Supplementary Fig. S3). These results suggest that PP242 activates PM H^+^-ATPase via pen-Thr phosphorylation in guard cells. PP242-induced phosphorylation of pen-Thr of PM H^+^-ATPase was also observed in *A. thaliana* leaf discs (Supplementary Fig. S4).

As PP242 induces stomatal opening and activates PM H^+^-ATPase, we evaluated the contribution of PM H^+^-ATPase activation by PP242 to stomatal opening through treatment with sodium orthovanadate (VD), an inhibitor of PM H^+^-ATPase (Gepstein et al. 1982, Kinoshita and Shimazaki 1999). Both light- and FC-induced stomatal opening were decreased by VD treatment, as was PP242-induced stomatal opening (Supplementary Fig. S5). These results suggest PM H^+^-ATPase involvement in PP242-induced stomatal opening.

### PP242-induced stomatal opening is unrelated to TOR and blue light signaling in guard cells

As PP242 has been reported as a TOR kinase inhibitor (Feldman et al. 2009) it may induce stomatal opening through TOR inhibition. To elucidate the molecular mechanism of PP242, we observed stomatal opening in response to PP242 in TOR-related mutants. Because homotypic *AtTOR* knockout mutation by transfer DNA insertion shows embryonic lethality (Deprost et al. 2007), we used a *TOR/tor* heterozygous knockout mutant. Chemical sensitivity is more pronounced when one allele is impaired, a phenomenon called haplo-insufficiency (Giaever et al. 1999). Studies have shown that chemical-induced inhibition of root elongation is more pronounced in *AtTOR/to*r heterozygous mutants than in wild-type (WT) plants, demonstrating haplo-insufficiency (Deprost et al. 2007, Montané and Menand 2013). In this study, *AtTOR/to*r heterozygous mutants displayed stomatal opening in response to PP242 and showed similar PP242 sensitivity to the *A. thaliana* Columbia-0 (Col-0; WT) plants (Supplementary Fig. S1C). We also used *raptor1(rb10)* mutants; RAPTOR1/RAPTORB is a component of the AtTOR complex in *A. thaliana* (Liu and Xiong 2022). The stomata of *rb10* mutants also opened in response to PP242 (Supplementary Fig. S1D). These data suggest that PP242-induced stomatal opening is not attributable to TOR inhibition.

Next, we examined the involvement of PP242 in blue light signaling using several mutants, including *phot1 phot2* (Kinoshita et al. 2001), *blus1* (Takemiya et al. 2013), and *bhp* (Hayashi et al. 2017). PP242-induced stomatal opening was normal in these mutants (Supplementary Fig. S6a–c). We also tested the effect of calyculin A (CA), an inhibitor of light-induced stomatal opening (Kinoshita and Shimazaki 1997), on PP242-induced stomatal opening. CA-treated stomata responded to PP242 and opened to a similar extent to non-CA-treated stomata (Supplementary Fig. S6d). These results suggest that the blue light signaling pathway is not related to PP242-induced stomatal opening.

### PP242 inhibits early ABA signal transduction in guard cells

**Figure 3.**
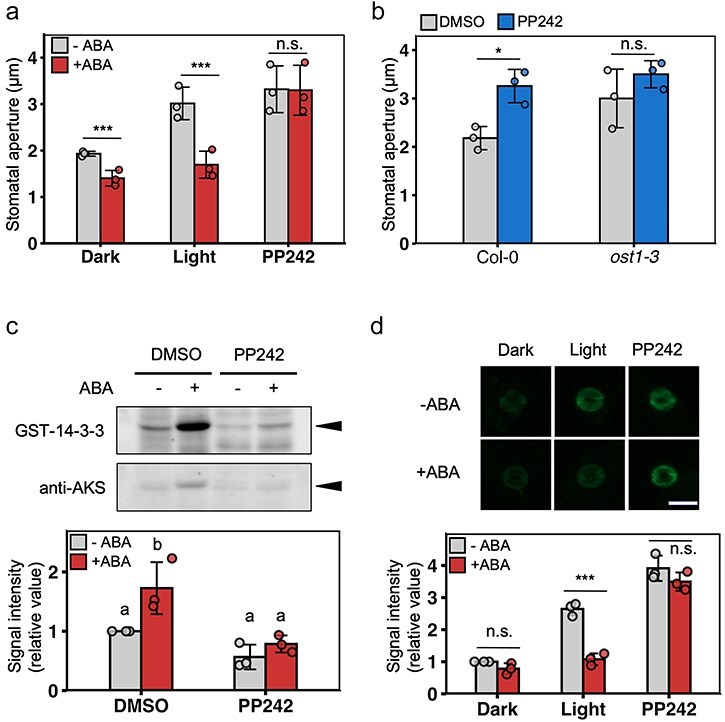
PP242 inhibits ABA signal transduction in guard cells. (a) Inhibition of ABA-induced stomatal closure by PP242. Epidermis samples from dark-adapted *A. thaliana* Col-0 were pretreated with DMSO, 50 µM PP242, or 10 µM FC in the dark or under red light treatment at 50 μmol/m^2^/s and blue light treatment at 10 μmol/m^2^/s for 1 h. Subsequently, DMSO or 20 µM ABA was added to the buffer and samples were incubated for 2 h. Data are mean ± SD (*n* = 3; 30 stomata per replicate). Asterisks indicate significant differences between −ABA and +ABA in each sample (***P < .001, n.s., not significant; Student’s *t*-test). (b) PP242-induced stomatal opening in the *ost1-3* mutant. Epidermis samples from *A. thaliana* WT or *ost1-3* were treated with 50 µM PP242 in the dark for 3 h. Data are mean ± SD (*n* = 3; 30 stomata per replicate). Asterisks indicate significant differences between DMSO and PP242 in each sample (*P < .05; n.s., not significant; Student’s *t*-test). (c) Inhibition of ABA-dependent phosphorylation of AKS1 by PP242. GCPs from *A. thaliana* Col-0 were pretreated with 50 µM PP242 in the dark for 1 h, and then treated with 20 µM ABA for 10 min. Far-western blotting was performed using the GST-14-3-3 probe. AKS1 detection by western blotting was performed using AKS antibody. Bar graph shows the signal intensity relative to the DMSO control. Data are mean ± SD (*n* = 3). Different letters indicate significant differences (*P *< .05; one-way ANOVA followed by Tukey’s HSD test). (d) Suppression of ABA-induced dephosphorylation of pen-Thr of PM H^+^-ATPase by PP242. Epidermis samples from *A. thaliana* Col-0 were treated with 50 μM PP242 in the dark or under red light treatment at 50 μmol/m^2^/s and blue light treatment at 10 μmol/m^2^/s for 30 min. Then, 20 μM ABA was added to the buffer and the mixture was incubated for 30 min. The bar graph shows fluorescence intensity. Data are mean ± SD (*n* = 3; fluorescence intensity of 50 stomata per replicate). Scale bar = 20 µm. Asterisks indicate significant differences between DMSO and ABA in each sample (***P < .001; n.s., not significant; Welch’s *t*-test).

Next, we investigated the involvement of PP242 in stomatal closing pathways, particularly the ABA signaling pathway. Mutants of ABA signal transduction components, such as *ost1-1, ost1-2*, and *abi1-1*, show wider stomatal pores opening than WT plants (Leymarie et al. 1998, Mustilli et al. 2002, Yoshida et al. 2002). The phosphorylation level of pen-Thr in PM H^+^-ATPase is higher in mutants of ABA signal components (Hayashi et al. 2011). As these phenotypes are similar to the effect of PP242, we investigated whether PP242 can inhibit ABA signal transduction in guard cells by examining the stomatal response to ABA. As reported previously (Jones and Mansfield 1970), stomata opened by light closed in response to ABA (Fig. 3a). However, stomata opened by PP242 did not close in response to ABA (Fig. 3a). This observation suggests the inhibition of ABA signal transduction by PP242 in guard cells.

As OST1 plays a hub role in ABA signal transduction in guard cells, we examined the effect of PP242 on stomatal aperture in *ost1-3* mutants. PP242-induced stomatal opening in *A. thaliana* WT plants, and *ost1-3* mutants showed wider stomatal apertures than those of WT plants before PP242 treatment, with no significant difference between dimethyl sulfoxide (DMSO)- and PP242-treated *ost1-3* plants (Fig. 3b). This result indicates that PP242 may have an inhibitory effect on ABA signal transduction, particularly on OST1.

To verify this possibility, we examined the activity of OST1 in PP242-treated GCPs of *A. thaliana*. AKSs are one of the direct substrates of OST1, and phosphorylated AKSs interact with 14-3-3 protein (Takahashi et al. 2013). Using this property, we detected OST1 activity via the binding of 14-3-3 protein to phosphorylated AKS1. Consistent with the previous report (Takahashi et al. 2013), ABA treatment significantly increased the interaction between 14-3-3 and AKS1 (Fig. 3c). Conversely, this interaction was not increased by ABA treatment in PP242-treated GCPs (Fig. 3c). Although the differences were nonsignificant, AKS1 showed decreased 14-3-3 binding in PP242 sample compared to DMSO sample even in the absence of ABA, indicating lower activity of OST1 (Fig. 3c).

In addition, we investigated the phosphorylation levels of PM H^+^-ATPase. ABA treatment inhibits the phosphorylation of pen-Thr in PM H^+^-ATPase in guard cells (Hayashi et al. 2011). As shown in Fig. 3d, ABA decreased the pen-Thr phosphorylation level in light-treated guard cells, but not in PP242-treated guard cells. Therefore, PP242 strongly inhibits the effects of ABA in guard cells, in terms of both stomatal closure and pen-Thr dephosphorylation in PM H^+^-ATPase.

### B3 clade Raf-like kinase is a target of PP242 in *A. thaliana*

**Figure 4.**
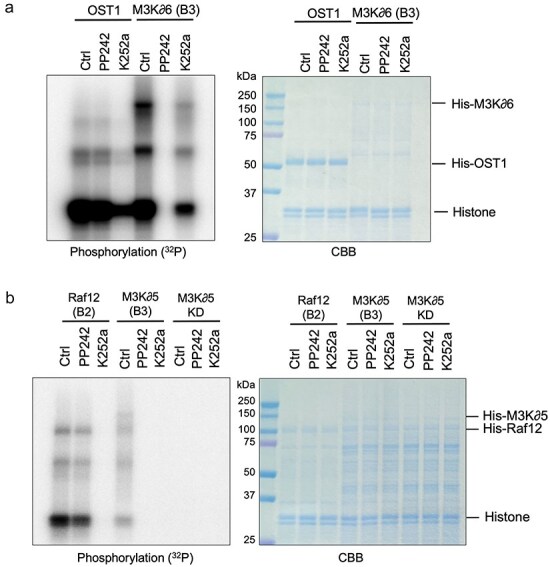
PP242 inhibits kinase activity in B3 clade Raf-like kinases *in vitro*. Kinase assay was conducted by using [γ-^32^P] ATP. (a) Recombinant His-OST1 and His-M3K∂6 proteins were incubated with 100 µM PP242 or 100 µM K252a for 20 min. A typical autoradiography image and a Coomassie brilliant blue staining image are shown (*n* = 3). (b) Recombinant His-Raf12, His-M3K∂5, and kinase dead (KD) His-M3K∂5 proteins were investigated. Experimental details are as described in (a) (*n* = 3).

As PP242 is a kinase inhibitor (Feldman et al. 2009), we focused on several kinases in early ABA signal transduction. Using recombinant proteins, we conducted protein phosphorylation assays with [γ-^32^P] ATP. In controls, His-OST1 autophosphorylated and phosphorylated histone proteins as a substrate (Fig. 4a). Kinase activity of His-OST1 was partially decreased by K252a, a potent kinase inhibitor, but not by PP242 (Fig. 4a). These results indicate that PP242 does not directly inhibit SnRK2 activity.

Upstream of subclass III SnRK2s including OST1, B2, and B3 clade Raf-like kinases phosphorylate SnRK2s (Katsuta et al. 2020, Takahashi et al. 2020, Lin et al. 2021, Soma et al. 2023). Therefore, we conducted protein phosphorylation assays using Raf-like kinases in both of these clades. Surprisingly, PP242 completely inhibited the kinase activity of His-M3K∂6 in B3 clade Raf-like kinases (Fig. 4a). Of this clade, M3K∂5 is the most highly expressed gene in guard cells (Hayashi et al. 2017). The His-M3K∂5 kinase activity was also inhibited by PP242 (Fig. 4b). M3K∂5 kinase dead form (KD) did not phosphorylate either itself or histones (Fig. 4b). Conversely, the kinase activity of Raf12, a B2 clade Raf-like kinase, was not inhibited by PP242 (Fig. 4b). Together, these results demonstrate that PP242 directly targets B3 clade Raf-like kinases *in vitro*.

**Figure 5.**
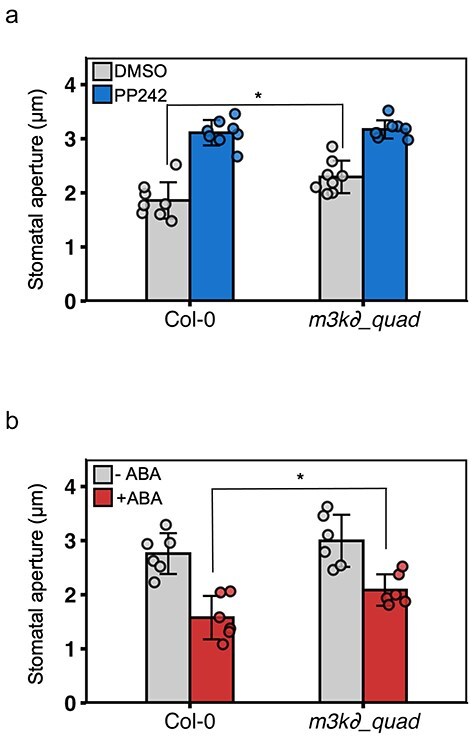
Stomatal apertures in the *m3k ∂1/∂6/∂5-2/∂7* B3 Raf quadruple mutant. (a) Stomatal apertures in the *m3k ∂1/∂6-1/∂5-2/∂7* mutant in response to PP242. Epidermis samples from *A. thaliana* Col-0 or *m3k ∂1/∂6-1/∂5-2/∂7* were treated with DMSO or 50 µM PP242 in the dark for 3 h. Data are mean ± SD (*n* = 8; 30 stomata per replicate). Asterisks indicate significant differences between DMSO samples in each plant (*P < .05; Student’s *t*-test). (b) ABA-induced stomatal closure in the *m3k ∂1/∂6-1/∂5-2/∂7* mutant. Epidermis samples were treated with red light at 50 μmol/m^2^/s and blue light at 10 μmol/m^2^/s for 2 h, and then treated with 20 µM ABA for 2 h under the same light condition. Data are mean ± SD (*n* = 6; 30 stomata per replicate). Asterisks indicate significant differences between ABA samples in each plant (*P < .05; Student’s *t*-test).

Finally, we investigated stomatal phenotypes in mutants of B3 clade Raf-like kinases. *A. thaliana* has six B3 clade Raf-like kinase genes. We measured stomatal aperture in *m3k∂1/∂6/∂5-2/∂7* quadruple mutants (Hsu et al. 2021b). The quadruple mutant showed somewhat wider stomatal pore opening compared to WT in the dark (Fig. 5a). PP242 induced stomatal opening in this mutant to levels comparable to those observed in WT (Fig. 5a). Next, we examined ABA-induced stomatal closure in this mutant. In the WT, stomata that had opened upon light exposure closed in response to ABA (Fig. 5b). Although this mutant also showed stomatal closure in response to ABA, these stomata showed slightly but significantly wider stomatal aperture compared to WT (Fig. 5b). This result is consistent with the previously reported B3 clade Raf-like kinases triple-mutant phenotype (Katsuta et al. 2020), and indicates that quadruple mutants of B3 clade Raf-like kinases show a partial but similar phenotype in PP242-treated stomata in WT.

## Discussion

**Figure 6.**
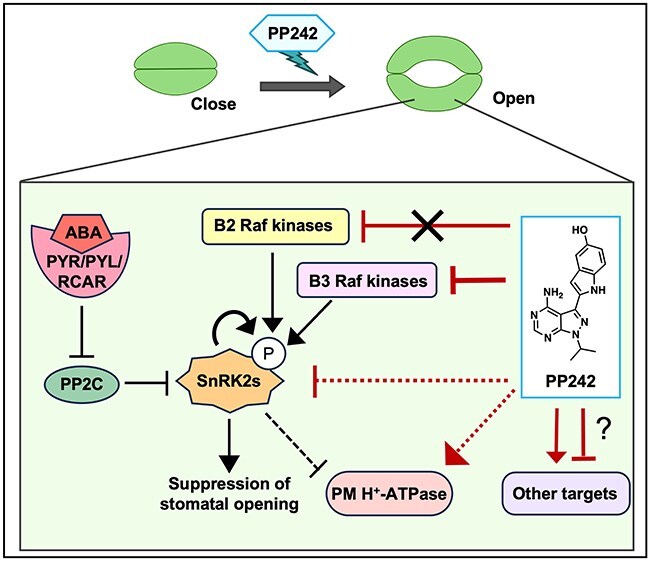
Proposed mechanism of PP242 effects in guard cells. In guard cells, PP242 strongly inhibits SnRK2s’ activity but does not directly inhibit SnRK2s. B3 clade Raf-like kinases were identified as targets of PP242.

In this study, we identified PP242 as a novel stomatal opening chemical and revealed its functional mechanism in guard cells (Fig. 6). PP242 induces pen-Thr phosphorylation in PM H^+^-ATPase, causing PM H^+^-ATPase activation. PP242 completely inhibits ABA-induced stomatal closure and the early ABA signaling pathway in guard cells. We also identified B3 clade Raf-like kinases as targets of PP242 in plants.

As PP242 is a TOR inhibitor, we analyzed the effect of TOR inhibition on stomatal apertures by testing 15 TOR inhibitors, including one examined in our previous study (Toh et al. 2018). Of these, only five chemicals, temsirolimus, everolimus, umirolimus, Torin2, and PP242, significantly induced stomatal opening (Fig. 1b, Supplementary Fig. S1a and b). As not all TOR inhibitors used in these experiments induced stomatal opening, stomatal opening induced by these five TOR inhibitors appears not to be caused by TOR inhibition. In addition, the *TOR/tor-4* heterozygous mutant and *rb10* mutant showed similar stomatal movement compared to the WT (Supplementary Fig. S1c and d). TOR is a widely conserved kinase in eukaryotes, and *RAPTOR* genes are also conserved in *A. thaliana* (Anderson et al. 2005). The *RAPTOR* gene, *RB10* co-regulates the function of *AtTOR* (Liu and Xiong 2022). The *rb10* mutant has been reported to have a wider stomatal aperture than the WT (Salem et al. 2018). However, in our experiment, the *rb10* mutant showed a phenotype similar to the WT in the dark, and showed normal stomatal opening in response to PP242 (Supplementary Fig. S1d). A recent study showed that TOR inhibition suppresses stomatal opening via the inhibition of starch degradation in guard cells (Han et al. 2022). Collectively, these results indicate that TOR plays a promotional role in stomatal opening, and that PP242-induced stomatal opening may not be caused by TOR inhibition.

Our experiments revealed the inhibition of ABA signaling by PP242 in guard cells (Figs. 3–4). However, there is a discrepancy between the functions of PP242 as a TOR inhibitor and as a stomatal opening inducer via ABA inhibition. TOR complexes and ABA signaling components function antagonistically in *A. thaliana* (Wang et al. 2018). TOR kinase suppresses the PYL ABA receptors through phosphorylation, as SnRK2 suppresses TOR function through RAPTOR1 phosphorylation (Wang et al. 2018). Given that PP242 is a TOR inhibitor, PP242 was expected to activate the ABA signaling pathway, as partially demonstrated in a previous study (Wang et al. 2018). However, we observed that PP242 strongly inhibited the ABA signaling pathway in guard cells (Fig. 3). Although this result contradicts previous findings, PP242 has a relatively high half-maximal inhibitory concentration compared to other TOR inhibitors (Montané and Menand 2019), such that the effect of PP242 in guard cells is likely attributable to off-target effects. A recent study showed that activated PM H^+^-ATPase increased TOR activity in plant cell suspensions (Primo et al. 2022). Therefore, TOR inhibition by PP242 may be partially offset by TOR activation due to the activation of PM H^+^-ATPase. We propose that the effect of PP242 on ABA signaling inhibition may surpass its inhibitory effect on TOR in guard cells.

PP242 completely inhibited early ABA signal transduction, including SnRK2.6/SRK2E/OST1 *in vivo* and B3 clade Raf-like kinases *in vitro* (Figs. 3c, 4a and b). In addition, PP242 had a partial effect on stomatal aperture in mutants of SnRK2.6/ SRK2E/OST1 (Fig. 3b). PP242 also suppressed ABA-induced pen-Thr dephosphorylation, leading to decreased PM H^+^-ATPase activity (Fig. 3d). Studies have demonstrated that mutants lacking ABA signal components, such as *abi1-1* and *abi2-1*, show wider stomatal opening even in the dark and higher pen-Thr phosphorylation levels than WT (Leymarie et al. 1998, Mustilli et al. 2002, Yoshida et al. 2002, Hayashi et al. 2011). Together, these results provide robust evidence that PP242 suppresses ABA signaling in guard cells, leading to stomatal opening and PM H^+^-ATPase activation. Our results strongly re-evaluate the importance of ABA function in guard cells under both drought and normal conditions.

FC has been known as a fungal toxin which induce intensive stomatal opening in plants. In this study, we newly identified PP242 as a novel stomatal opening inducer. FC has directly increase activities of transporters such as PM H^+^-ATPase and KAT1 (Saponaro et al. 2017). PP242 has a similarity in increasing phosphorylation level of PM H^+^-ATPase. On the other hand, PP242 also affects ABA signal transduction. As far as we know, there is no report that FC inhibits early ABA signaling components including SnRK2s although FC suppresses ABA-induced stomatal closure (Huang et al. 2014). Further investigation is needed to clarify the effect of FC on early ABA signaling components.

PP242 was developed as an mTOR inhibitor (Feldman et al. 2009). In this study, we aimed to identify the targets of PP242 in plants and revealed B3 clade Raf-like kinases as PP242 targets (Fig. 4a and b). PP242 did not directly inhibit OST1 but rather completely inhibited the kinase activity of B3 clade Raf-like kinases *in vitro* (Fig. 4a and b). The B3 clade Raf-like kinases phosphorylate and activate SnRK2s both in the ABA and the osmotic stress signaling pathways in *A. thaliana* (Katsuta et al. 2020, Takahashi et al. 2020, Lin et al. 2021) and B2 clade Raf-like kinases continually phosphorylate SnRK2s (Soma et al. 2023). As PP242 strongly inhibits the activity of SnRK2s *in vivo*, PP242 was anticipated to also inhibit B2 clade Raf-like kinases in this study. Contrary to our expectations, PP242 did not exhibit a strong inhibitory effect on B2 clade Raf-like kinase Raf12 *in vitro* (Fig. 4b). We genetically validated the mechanism of B3 clade Raf-like kinases in guard cells using quadruple mutants in *A. thaliana*, which showed slightly but significantly wider stomatal aperture openings under DMSO treatment and weaker but significant sensitivity under ABA treatment (Fig. 5a and b), corresponding with previous findings (Katsuta et al. 2020, Hsu et al. 2021b). Based on these results, we could not conclude the complete contribution of B3 clade Raf-like kinases to ABA signaling in guard cells, although this may be attributable to genetic redundancy. The generation of plants that specifically suppress B3 clade Raf-like kinases in guard cells will elucidate the importance of these kinases in guard cells. Our results provide another possible explanation; PP242 may have other targets related to signal transduction of stomatal movements (Fig. 6). Further investigation, including target identification through chemical pulldown, will clarify this possibility and provide deeper insights into crosstalk related to physiological functions.

In conclusion, we identified a novel stomatal opening inducer, PP242, which also functions as an activator of PM H^+^-ATPase in guard cells and mesophyll cells via pen-Thr phosphorylation in PM H^+^-ATPase. The molecular function of PP242 in guard cells is the inhibition of the early ABA signaling pathway. Further investigations using PP242 could contribute to a better understanding of ABA signaling under normal conditions. We identified effects of PP242 in several species, including *C. benghalensis, A. thaliana*, and *V. faba*, having potential to be used as a novel stomatal opening inducer, as well as FC. Therefore, the chemical manipulation of stomatal aperture function is anticipated to have broad agricultural applications.

## Materials and Methods

### Plant growth conditions

The *A. thaliana* Columbia-0 (Col-0) ecotype was used as a background in this study. *Arabidopsis thaliana* plants were grown in soil under long-day conditions (16 h white light, 50 µmol/m^2^/s light intensity/8 h dark) with a relative humidity of 55–75% in a growth room. The *m3k∂1//∂5-2/∂6-1/∂7* quadruple mutant was used in a previous study (Hsu et al. 2021b). The *ost1-3* mutant was obtained from the Arabidopsis Biological Resource Center (Yoshida et al. 2002). *Commelina benghalensis* and *Vicia faba* cv. ‘Ryosai Issun’ plants were grown as previously described (Toh et al. 2018).

### Stomatal measurements

*Arabidopsis thaliana* rosette leaves from 4- to 6-week-old plants were used for stomatal measurements, which were conducted according to previous method (Inoue et al. 2008), with modifications. Rosette leaves from dark-adapted plants were blended in 39 ml of Milli-Q water twice using a blender (Waring Commercial, Stamford, CT, USA) in high mode for 3 s. In each experiment, 30 stomata were measured with light microscopy. Stomatal measurements were always performed in the afternoon.

Epidermis from dark-adapted *C. benghalensis* was isolated from the adaxial side of the leaf using tweezers. The epidermis samples were floated on stomatal opening buffer (MES-bistrispropane [pH 6.5], 50 mM KCl, and 0.1 mM CaCl_2_) and treated with agents for 3 h in the dark. In each experiment, 30 stomata were measured with light microscopy.

### Immunostaining

An immunostaining assay was performed as previously described (Aihara et al. 2023), with modifications. Epidermis samples were obtained from dark-adapted *A. thaliana* rosette leaves by blending three times in high mode for 4 s. In the time-course experiment, the 0-min treatment consisted of treating the epidermis with an equal volume of DMSO for 5 min. In the first antibody treatments, anti-pen-pThr antibody was used to detect the phosphorylation levels of PM H^+^-ATPase, as previously described (Hayashi et al. 2010). Anti-cat antibody was used to detect protein amounts in PM H^+^-ATPase as previously described (Hayashi et al. 2010). Captured images were quantified using Fiji software (National Institutes of Health, Bethesda, MD, USA).

### Isolation of GCPs from *A. thaliana* and *V. faba*

The isolation of GCPs from *A. thaliana* was performed as previously described (Hayashi et al. 2024) and that from *V. faba* was conducted according to a different method (Kinoshita and Shimazaki 1999), with modifications. Epidermis samples from *V. faba* were treated with an enzyme solution consisting of 1% (w/v) Cellulase Onozuka RS (Yakult Honsha Co., Tokyo, Japan), 0.5% (w/v) Macerozyme R-10 (Yakult Honsha Co.), 0.25 M mannitol, 0.2% (w/v) bovine serum albumin (BSA), and 1 mM CaCl_2_ (pH 5.5) for 12 min at 27°C. A second digestion was performed in a solution consisting of 2% (w/v) Cellulase Onozuka RS, 0.5% (w/v) Macerozyme R-10, 0.4 M mannitol, 0.2% (w/v) BSA, and 1 mM CaCl_2_ (pH 5.5) for 1 h at 27°C. After enzyme treatment, the solution was passed through a 30 μm mesh and the filtrate was centrifuged at 120 × *g* for 7 min at 4°C. The precipitated GCPs were suspended in a solution consisting of 0.4 M mannitol and 1 mM CaCl_2_, and centrifuged twice at 120 × *g* for 7 min at 4°C.

### Western blotting of GCPs from *V. faba*

GCPs isolated from *V. faba* were suspended in GCP suspension buffer [5 mM MES-NaOH (pH 6.0), 10 mM KCl, 0.4 M mannitol, and 1 mM CaCl_2_] and treated in the dark for 1 h on ice. These GCPs were treated with reagents and collected by centrifugation, and then the precipitated GCPs were suspended in GCP disruption buffer consisting of 10 mM MOPS-KOH (pH 7.5), 2.5 mM ethylenediaminetetraacetic acid (EDTA), 1 mM phenylmethylsulfonyl fluoride (PMSF), 20 µM leupeptin, and 1/30 units µL^−1^ DNase I recombinant (no. 4536282001; Roche, Basel, Switzerland). Then, 0.5% (v/v) Triton X-100 was added to the sample and solubilization buffer **[**2% sodium dodecyl sulfate (SDS), 1 mM EDTA, 20% glycerol, 10 mM Tris-HCl (pH 6.8), 2.5 mM NaF, 1 mM PMSF, 20 µM leupeptin, and 80 mM dithiothreitol (DTT)] was added to achieve a sample:solubilization buffer ratio of 8:5.

Protein samples were then separated with SDS–polyacrylamide gel electrophoresis (PAGE) and transferred onto a nitrocellulose membrane. The membrane was treated with blocking buffer containing 5% skim milk in TTBS [0.05% (w/v) Tween-20, 20 mM Tris-HCl (pH 7.4), and 140 mM NaCl], for 30 min. The membrane was treated with a primary antibody overnight at 4°C. Anti-pen-pThr antibody was used to detect the phosphorylation levels of *V. faba* H^+^-ATPase (VHA), as previously described (Kinoshita and Shimazaki 1999). Anti-VHA antibody was used to detect the VHA protein level. Membranes were washed with TTBS and then treated with goat antirabbit IgG horseradish peroxidase (HRP) antibody (no. 170–6515; Bio-Rad, Hercules, CA, USA), diluted 1:3000 in blocking buffer for 3 h at room temperature, and then washed with TTBS prior to signal detection. Signal intensity was quantified using Fiji software.

### H^+^ pumping measurements

Measurements of H^+^ pumping by *V. faba* GCPs were conducted as previously described (Shimazaki et al. 1992). GCPs were suspended in H^+^ pumping buffer [0.125 mM MES-NaOH (pH 6.0), 1 mM CaCl_2_, 0.4 M mannitol, and 10 mM KCl]. The GCP suspension was treated with red light at 600 µmol/m^2^/s for at least 30 min, until the pH stabilized. Then, 50 µM PP242 was added to the buffer and the pH change was measured. Finally, the amount of H^+^ released was calculated as the decrease in pH with 5 µl of 1 mM HCl (corresponding to 5 nmol H^+^).

### Western blotting and far-western blotting in *A. thaliana* GCPs

*Arabidopsis thaliana* GCPs were suspended in GCP buffer [5 mM MES-NaOH (pH 6.0), 10 mM KCl, 0.2 M mannitol, and 0.5 mM CaCl_2_] and treated with reagents. GCPs were then centrifuged and frozen in liquid N_2_. SDS solution [1.82% SDS, 0.91 mM EDTA, 18.2% glycerol, 9.1 mM Tris-HCl (pH 6.8), 0.01% CBB, 2.5 mM NaF, 1 mM PMSF, 20 µM leupeptin, and 80 mM DTT] was added to the GCPs. The samples were heated at 95°C for 3 min and then were separated using SDS–PAGE and transferred onto a nitrocellulose membrane. The membrane was treated with blocking buffer.

To detect interactions between phosphorylated AKS1 and 14-3-3, far-Western blotting was conducted as previously described (Takahashi et al. 2013). Briefly, 0.25 µM GST-14-3-3 phi (Kinoshita and Shimazaki 1999) in blocking buffer was used as a probe and incubated overnight at 4°C. The membrane was washed with TTBS and treated with GST antibody (Takahashi et al. 2013), diluted 1:3000 in blocking buffer for 2 h at room temperature, and then washed again and treated with goat antirabbit IgG HRP antibody, diluted to 1:3000 in blocking buffer for 2 h at room temperature.

To detect AKS1 protein, membranes used in the far-Western blot were reprobed. The membrane was washed with Milli-Q water and treated with reprobing solution (7 m guanidine hydrochloride, 50 mM glycine, 0.05 mM EDTA-2Na, 0.1 M KCl, and 20 mM 2-mercaptoethanol) for 10 min, and then washed with TTBS and treated with blocking buffer. The membrane was then treated with AKS-antibody (Takahashi et al. 2013), diluted to 1:3000 in blocking buffer overnight at 4°C, washed, treated with goat anti-rabbit IgG HRP antibody, and diluted to 1:3000 in blocking buffer for 2 h at room temperature. Signal intensity was quantified using Fiji software.

### *In vitro* kinase assay

All proteins used in this experiment were expressed in *Escherichia coli*. Plasmids of 6 × His-OST1 and 6 × His-M3K∂6 were described in a previous study (Takahashi et al. 2020). The M3K∂5 (AT4G24480.2) insert was amplified from cDNA of *A. thaliana* Col-0 rosette leaves. The KD version of M3K∂5 was constructed using 6 × His-M3K∂5 as a template. To amplify the Raf12 insert, Col-0 rosette leaf epidermis samples were treated with 50 µM ABA for 3 h in stomatal opening buffer. RNA isolated from this sample was used to construct cDNA using the primers listed in Supplementary Table S1. We used pET30a (+) as a vector, and generated plasmids through In-Fusion cloning (TaKaRa Bio, Kusatsu, Japan). The constructed plasmids were introduced into *E. coli* strain BL21 (DE3). Protein expression was induced by the addition of isopropyl β-d-thiogalactopyranoside. His-tagged proteins were purified using Ni-charged Profinity IMAC Resin (Bio-Rad) and eluted with elution buffer [50 mM Tris-HCl (pH 8.0), 300 mM NaCl, and 250 mM imidazole].

His-tagged proteins were treated in reaction buffer [50 mM Tris-HCl (pH 7.5), 10 mM MgCl_2_, 1 mM DTT, 0.1% Triton X-100, and 1 µg histone] for 20 µl scale reactions. Next, 100 µM PP242, 100 µM K-252a, or an equal volume of DMSO were added to the buffer and the samples were incubated for 20 min at room temperature. The phosphorylation reaction was initiated by the addition of 200 µM cold ATP and 1 µCi [γ-^32^P] ATP. After 30 min of incubation at room temperature, 10 µl of 3 × SDS buffer [30 mM Tris-HCl (pH 8.0), 45% (w/v) sucrose, 3% (w/v) SDS, 3 mM EDTA, 7.5% (w/v) 2-mercaptoethanol, and 0.06% (w/v) CBB] was added and the samples were heated at 95°C for 3 min. Protein samples were then separated with SDS–PAGE. The acrylamide gels were fixed for 30 min and then washed with Milli-Q water and stained with CBB. The signal from the gel was detected with a Storage phosphor screen (BAS-IP MS 2025, Fujifilm).

### Compounds

All compounds used in this study were obtained commercially (Supplementary Table S2). DMSO was used as the solvent for PP242, and when adding PP242 to the butter, the buffer was added in one go and vigorously mixed to prevent crystallization.

### Statistical analysis

All statistical analyses were performed using R v4.4.0 and R Studio v2024.04.1.

## Supplementary Material

pcaf013_Supp

## Data Availability

All data that support the findings of this study are included in the manuscript and Supplementary Data of this article. The raw data in this study are available from the corresponding author upon request.
