# Supplementary material for: Identification of a novel stomatal opening chemical, PP242, that inhibits early abscisic acid signal transduction in guard cells
Source: Plant Cell Physiol. 2025 Jan 30;66(6):854–65. doi: 10.1093/pcp/pcaf013 (PMC12290280; doi:10.1093/pcp/pcaf013)
Supplement: pcaf013_Supp [file pcaf013_supp.zip › pcaf013_Supp/suppl_data/pcp-2024-e-00241-File008.pdf]

Lists of Supplemental Data

Supplemental materials and methods
Supplemental Figure S1. Target of rapamycin (TOR) inhibition may not cause PP242-induced stomatal opening.
Supplemental Figure S2. PP242 induces stomatal opening in several species.
Supplemental Figure S3. PP242-dependent H^+ pumping in *V. faba* guard cell protoplasts.
Supplemental Figure S4. P242 induces the phosphorylation of pen-Thr of PM H^+-ATPase in leaf discs.
Supplemental Figure S5. Plasma membrane (PM) H^+-ATPase activation is important for PP242-induced stomatal opening.
Supplemental Figure S6. PP242 induces stomatal opening in blue light signaling-related mutants.
Supplemental Table S1. Primers used in this study.
Supplemental Table S2. Compounds used in this study.

Supplemental Materials and Methods

Plant growth conditions

Plant growth conditions were as described in the main text (Materials and Methods). The *bhp-1* mutant, *TOR/tor-4* hetero mutant, and *rb10* (*raptor1b*) mutant were described in previous studies (Hayashi et al. 2017, Salem et al. 2018, Toh et al. 2018), and *gl1* was used as the background ecotype of *phot1-5 phot2-1* (Kinoshita et al. 2001). *Arabidopsis thaliana* Col-0 was the background ecotype of *blus1-3* (Takemiya et al. 2013), *bhp-1* (Hayashi et al. 2017), *TOR/tor-4*, and *rb10*. Seeds of the *blus1-3* mutant (SALK_000221) and *rb10* mutant (SALK_101990) were obtained from the Arabidopsis Biological Resource Center.

Stomatal measurements in *Vicia faba*

Epidermis samples from dark-adapted *V. faba* were isolated from the adaxial side of the leaf in water using tweezers. Epidermis samples were floated in stomatal opening buffer consisting of MES-bistrispropane (BTP) (pH 6.5), 50 mM KCl, and 0.1 mM $CaCl_2$, and treated with the indicated reagents in the dark for 3 h. In each sample, 30 stomata were measured under light microscopy.

Western blotting of *A. thaliana* leaf discs

Leaf discs were isolated form dark-adapted *A. thaliana* rosette leaves using a biopsy punch, and then filtrated with stomatal opening buffer 30 times and treated with reagents in the dark for 1 h. The leaf discs were then frozen with liquid N_2 and homogenized using a pestle. SDS solution (1.82% SDS, 0.91 mM ethylenediaminetetraacetic acid, 18.2% glycerol, 9.1 mM Tris-HCl, 0.01% Coomassie brilliant blue, 2.5 mM NaF, 1 mM phenylmethylsulfonyl fluoride, 20 μM leupeptin, and 80 mM dithiothreitol) was added to the sample. Sample buffers were centrifuged at 10,000 × *g* for 10 min at room temperature.

Supernatant protein samples were separated via SDS–polyacrylamide gel electrophoresis and transferred onto a nitrocellulose membrane. The membrane was treated with blocking buffer for 30 min, and then treated with a primary antibody overnight at 4°C. Anti-pen-pThr antibody and anti-cat antibody were then used. The membrane was washed with TTBS and treated with goat anti-rabbit IgG horseradish peroxidase antibody, diluted to 1:3000 in blocking buffer for 2 h at room temperature, and washed with TTBS. Signals were detected and their intensity was quantified using Fiji software.

Stomatal measurement with sodium orthovanadate (VD) treatment

Epidermis samples from dark-adapted *Commelina benghalensis* were isolated from the adaxial side of the leaf using tweezers and then floated in buffer consisting of MES-BTP (pH 6.5), 10 mM KCl, and 0.1 mM $CaCl_2$. Epidermis samples were pre-treated with VD for 20 min, and then treated with reagents in the dark or under white light for 3 h. In each sample, 30 stomata were measured under light microscopy.

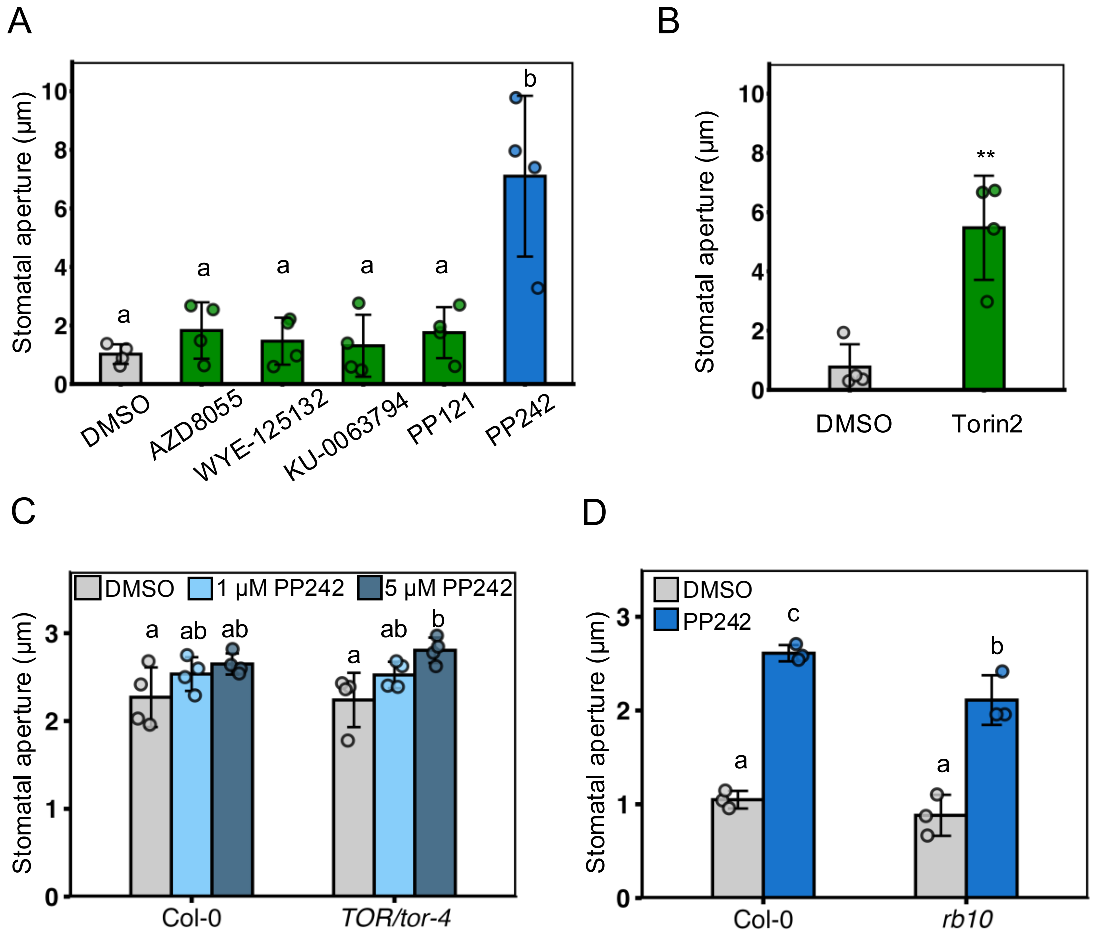

Supplementary Fig. S1 Target of rapamycin (TOR) inhibition may not cause PP242-induced stomatal opening. (A) Effect of TOR inhibitors on stomatal opening in *Commelina benghalensis*. Dark-adapted epidermis samples were treated with 50 μM of the indicated chemicals in the dark for 3 h. Data are the means ± standard deviation (SD; n = 4; 30 stomata per replicate). Different letters indicate significant differences ($P < 0.05$; one-way analysis of variance [ANOVA] followed by Tukey's honest significant difference [HSD] test). (B) Effect of Torin2, a TOR inhibitor, on stomatal opening in *C. benghalensis.* Dark-adapted epidermis samples were treated with 50 μM Torin2 in the dark for 4 h. Data are the means ± SD (n = 4; 30 stomata per replicate). Asterisks indicate significant differences between dimethyl sulfoxide (DMSO) and Torin2 (**$P < 0.01$; Welch's *t*-test). (C) PP242-dependent stomatal opening in *A. thaliana* Col-0 and *TOR/tor-4* plants. Dark-adapted epidermis samples were treated with PP242 in the dark for 3 h. Data are the means ± SD (n = 4; 45 stomata per replicate). Different letters indicate significant differences ($P < 0.05$; one-way ANOVA followed by Tukey's HSD test). (D) PP242-dependent stomatal opening in *A. thaliana* Col-0 and *rb10* plants. Dark-adapted epidermis samples were treated with 50 μM

PP242 in the dark for 3 h. Data are the means ± SD (n = 3; 30 stomata per replicate). Different letters indicate significant differences ($P < 0.05$; one-way ANOVA followed by Tukey's HSD test).

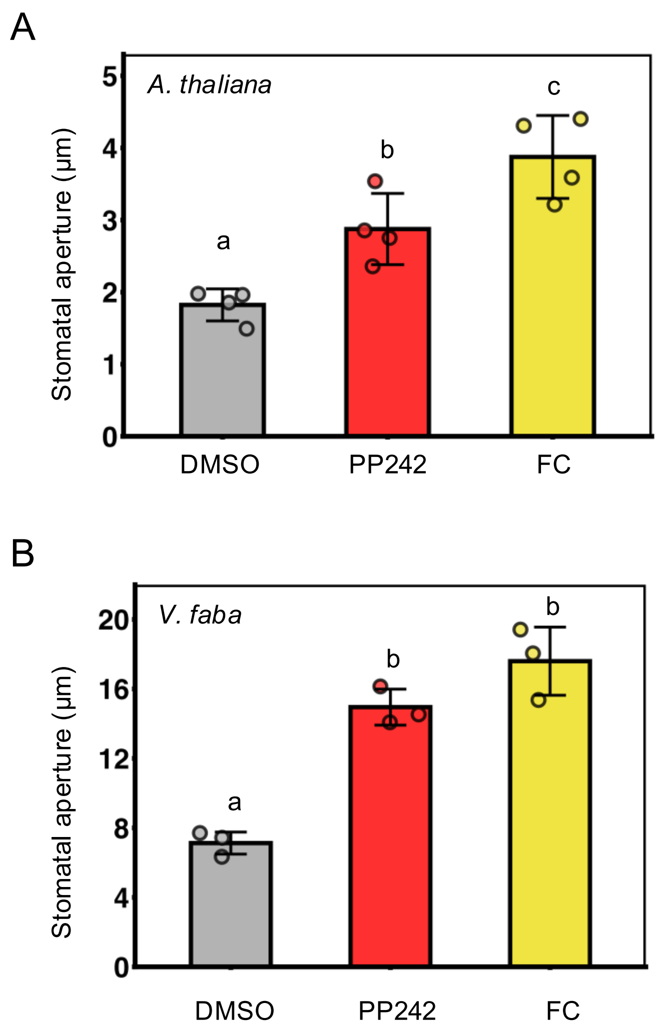

Supplementary Fig. S2 PP242 induces stomatal opening in several species. (A) PP242-induced stomatal opening in *A. thaliana* Col-0. Dark-adapted epidermis samples were treated with 50 μM PP242 or 10 μM fusicoccin (FC) in the dark for 3 h. Data are the means ± SD (n = 4; 30 stomata per replicate). Different letters indicate significant differences ($P < 0.05$; one-way ANOVA followed by Tukey's HSD test). (B) PP242-induced stomatal opening in *Vicia faba*. Dark-adapted epidermis samples were treated with 50 μM PP242 or 10 μM FC in the dark for 3 h. Data are the means ± SD (n = 3; 30 stomata per replicate). Different letters indicate significant differences ($P < 0.05$; one-way ANOVA followed by Tukey's HSD test).

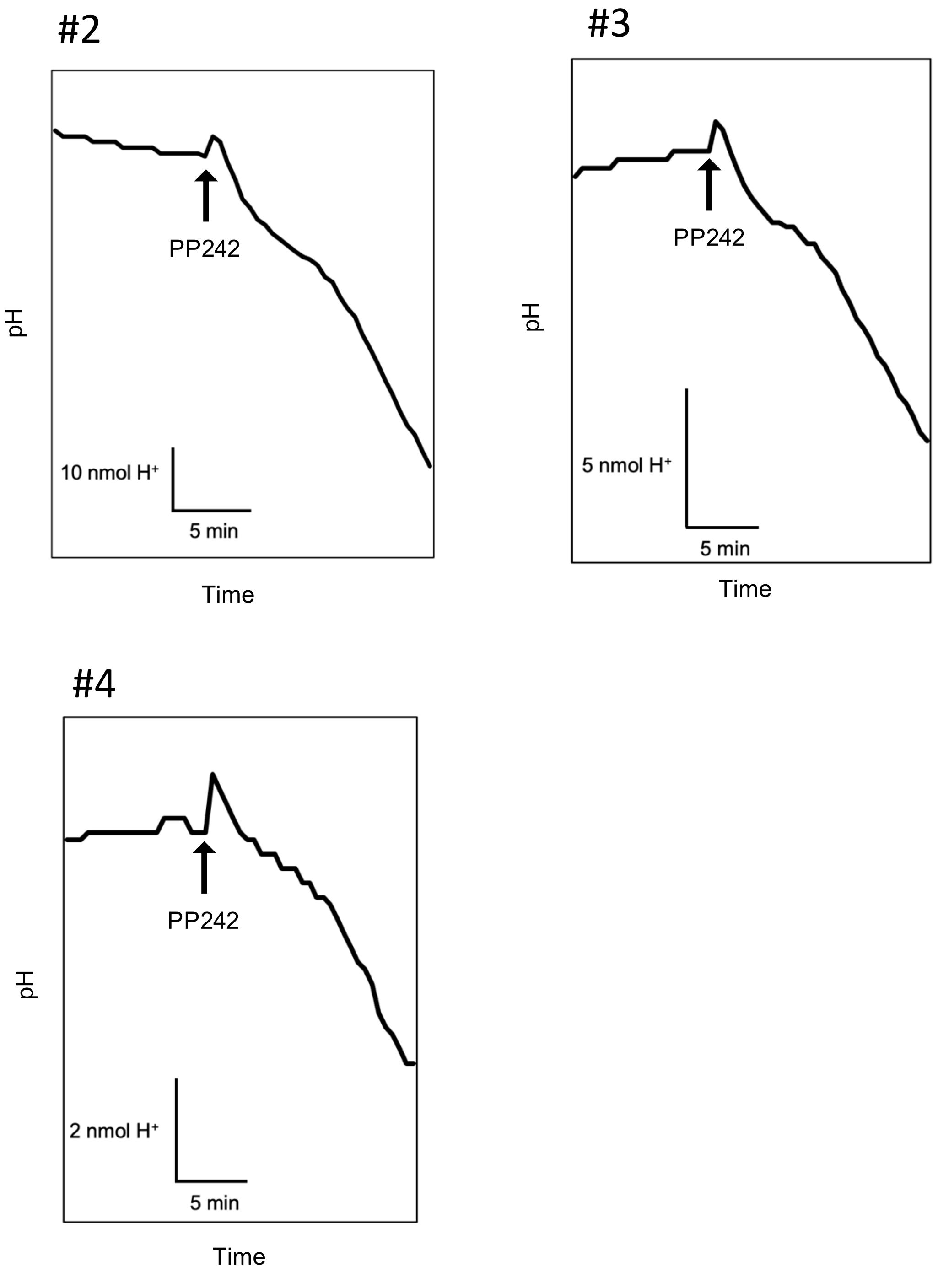

Supplementary Fig. S3 PP242-dependent H^+ pumping in *V. faba* guard cell protoplasts. Experiments were conducted as described for Fig. 2C. Data from three of the four experiments are shown.

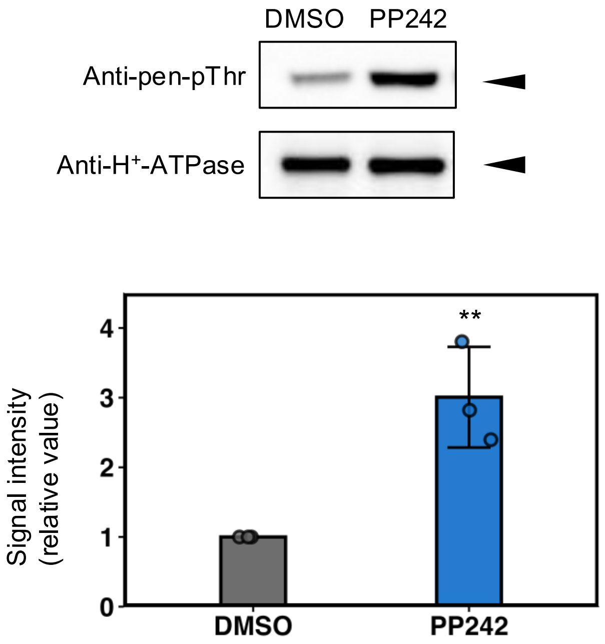

Supplementary Fig. S4 PP242 induces the phosphorylation of pen-Thr of PM H^+-ATPase in leaf discs. Typical (upper) immunoblot images and (lower) band intensity quantification results are shown. Leaf discs from dark-adapted rosette leaves of *A. thaliana* Col-0 were treated with 50 μM PP242 in the dark for 1 h. Immunoblotting was performed using individual antibodies. H^+-ATPase phosphorylation levels were determined by the band intensity ratio compared to controls. Data are the means ± SD (n = 3). Asterisks indicate significant differences ($P < 0.01$; Welch's *t*-test).

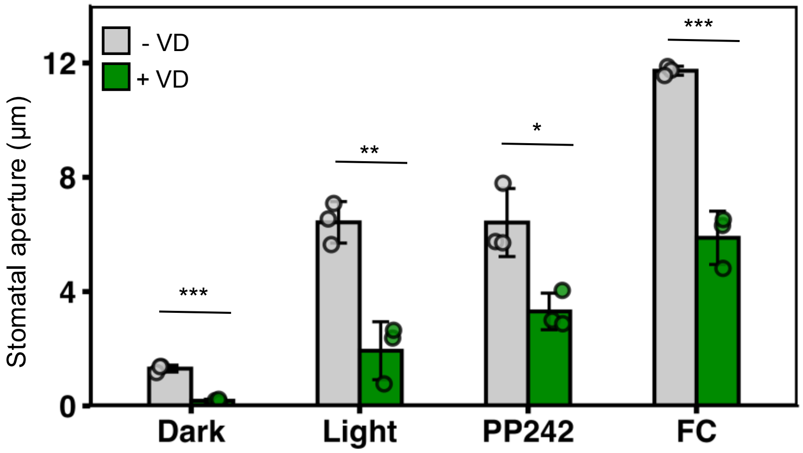

Supplementary Fig. S5 Plasma membrane **(**PM) H^+-ATPase activation is important for PP242-induced stomatal opening. Epidermis samples from dark-adapted *C. benghalensis* were pre-treated with or without 1 mM sodium orthovanadate (VD) for 20 min, and then treated with 50 μM PP242 or 10 μM FC in the dark for 3 h. For light exposure, epidermis samples were treated with white light at 50 μmol m^{-2} s^{-1} for 3 h. Data are the means ± SD (n = 3; 30 stomata per replicate). Asterisks indicate significant differences between −VD and +VD in each sample (*P < 0.05; **P < 0.01; ***P < 0.001; Student's *t*-test).

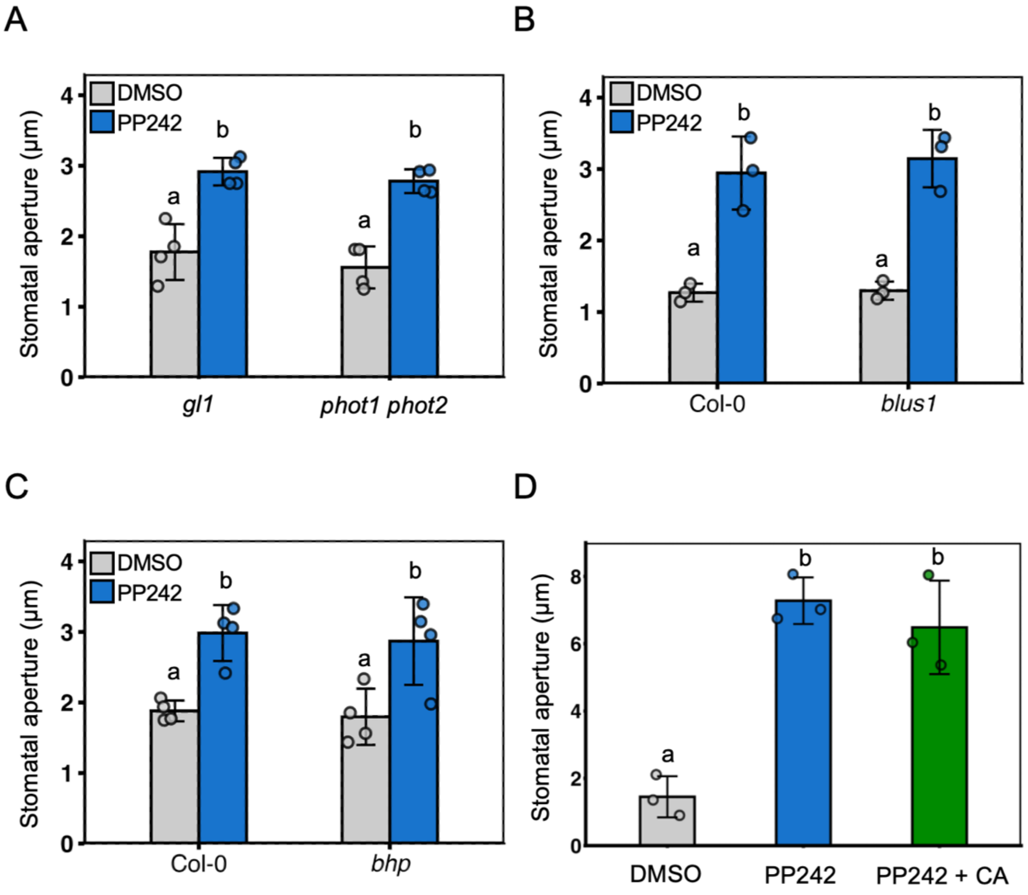

Supplementary Fig. S6 PP242 induces stomatal opening in blue light signaling-related mutants. (A) PP242 induced stomatal opening in epidermis samples from *A. thaliana gl1* and *phot1 phot2*. Epidermis samples from dark-adapted plants were treated with 50 μM PP242 in the dark for 3 h. Data are the means ± SD (n = 4; 30 stomata per replicate). Different letters indicate significant differences ($P < 0.05$; one-way ANOVA followed by Tukey's HSD test). (B) PP242 induced stomatal opening in epidermis samples from *A. thaliana* Col-0 and *blus1* plants. Experimental details and statistical methods were as described for (A) (n = 3). (C) PP242 induced stomatal opening in epidermis samples from *A. thaliana* Col-0 and *bhp* plants. Experimental details and statistical methods are as described for (A) (n = 4). (D) Effects of calyculin A (CA) on PP242-induced stomatal opening. Epidermis samples from dark-adapted *C. benghalensis* were pre-treated with 1 μM CA for 20 min, and then treated with 50 μM PP242 in the dark for 3 h. Data are the means ± SD (n = 3; 30 stomata per replicate). Different letters indicate significant differences ($P < 0.05$; one-way ANOVA followed by Tukey's HSD test).

Supplementary Table S1 Primers used in this study.

**Primers**	**Sequence (5' → 3')**
M3K∂5_CDS1st_Fw	TCTCCAGTATCTGGCGACG
M3K∂5_CDS1st_Rv	ACAGTGTTTCTGTTAGCACCG
pET_M3K∂5_In-Fusion_Fw	GCTGATATCGGATCCGAATTCATGCCTCACCGGACTAC
pET_M3K∂5_In-FusionF_Rv	TTGTCGACGGAGCTCGAATTCTATAGTATGGGAGCTGATT
M3K∂5_D812N_KD_Fw	ACAGTGAAGGTTTGCAATTTTGGACTTTCAAGATTC
M3K∂5_D812N_KD_Rv	GCAAACCTTCACTGTCCAGTTCTTATCAACCAGTAG
pET_Raf12_In-Fusion_Fw	TATCGGATCCGAATTCATGGCCGGAAACAACTCG
pET_Raf12_In-Fusion_Rv	GACGGAGCTCGAATTTCAATCGTCTTCTTCTTGCG

Supplementary Table S2 Compounds used in this study.

Compound	Source
PP242	Selleck Chemicals
Temsirolimus	Selleck Chemicals
Fusicoccin	Sigma (Merck)
(+/−) Abscisic acid	Sigma (Merck)
Calyculin A	FUJIFILM
K252a	Funakoshi Co. Ltd.
AZD8055	Funakoshi Co. Ltd.
WYE-125132	Sigma (Merck)
KU-0063794	CEM Corp.
PP121	Selleck Chemicals
Torin2	Sigma (Merck)
Sodium orthovanadate	Sigma (Merck)
